# Sterol 27-hydroxylase gene dosage and the antiatherosclerotic effect of Rifampicin in mice

**DOI:** 10.1042/BSR20171162

**Published:** 2018-01-25

**Authors:** Line Zurkinden, Dmitri Sviridov, Bruno Vogt, Geneviève Escher

**Affiliations:** 1 Department of Nephrology, Hypertension and Clinical Pharmacology, Department of Clinical Research, Inselspital, University of Bern, Switzerland; 2 Baker IDI Heart and Diabetes Institute, PO Box 6492, Melbourne, VIC 3004, Australia

**Keywords:** ApoE knock out mice, 27-hydroxycholesterol, Western diet

## Abstract

Sterol 27-hydroxylase (CYP27A1) catalyzes the hydroxylation of cholesterol to 27-hydroxycholesterol (27-OHC) and regulates cholesterol homeostasis. In *Cyp27a1/ Apolipoprotein E* (*ApoE*) double knockout (KO) mice fed with Western diet (WD), the atherosclerotic phenotype found in *ApoE* KO mice was reversed. As protective mechanism, up-regulation of *Cyp3a11* and *Cyp7a1* was proposed. *Cyp27a1* heterozygote/*ApoE* KO (het) mice, with reduced *Cyp27a1* expression and normal levels of *Cyp7a1* and *Cyp3a11*, developed more severe lesions than *ApoE* KO mice. To analyze the contribution of *Cyp3a11* to the protection of atherosclerosis development, *Cyp3a11* was induced by Rifampicin (RIF) in *ApoE* KO and het mice. Males were fed with WD and treated daily with RIF (10 mg/kg ip) or vehicle for 4 weeks. Atherosclerosis was quantified in the aortic valve. Plasma lipids and 27-hydroxycholesterol (27-OHC), expression of cytochromes P450 and genes involved in cholesterol transport and bile acids (BAs) signaling in liver and intestine, and intestinal cholesterol absorption were analyzed. RIF increased expression of hepatic but not intestinal *Cyp3a11* 4-fold in both genotypes. In *ApoE* KO mice treated with RIF, we found a 2-fold decrease in plasma cholesterol, and a 2-fold increase in high-density lipoprotein/low-density lipoprotein ratio and CY27A1 activity. Intestinal cholesterol absorption remained unchanged and atherosclerotic lesions decreased approximately 3-fold. In het mice, RIF had no effect on plasma lipids composition, CYP27A1 activity, and atherosclerotic plaque development, despite a reduction in cholesterol absorption. In conclusion, the antiatherogenic effect of *Cyp3a11* induction by RIF was also dependent on *Cyp27a1* expression.

## Introduction

Sterol 27-hydroxylase (CYP27A1) is a mitochondrial cytochrome P450 enzyme which hydroxylates cholesterol at C27 into 27-hydroxycholesterol (27-OHC) and cholestenoic acid [1,2]. CYP27A1 catalyzes the first step of the classic and intermediate step of the alternative pathway of bile acid (BA) biosynthesis [3,4]. It is also involved in cholesterol efflux, the first and rate limiting step of reverse cholesterol transport (RCT). RCT removes cholesterol from extrahepatic tissues to high-density lipoprotein (HDL) and transports it to the liver for elimination via BA. Overexpression of CYP27A1 in CHOP cells or macrophages stimulates efflux [5,6]. In *in vivo* RCT studies, more [^3^H]-cholesterol is delivered to the liver and feces following injection of macrophages transfected with a combination of genes containing *Cyp27a1* [7]*. In vitro*, 27-OHC acts as a negative feed-back regulator of the rate limiting enzyme of cholesterol biosynthesis, the enzyme 3-hydroxy-3-methylglutaryl-CoA reductase (HMGCR) [8]. Thus, CYP27A1 is a substantial modulator of cholesterol homeostasis, owing its participation in pathways regulating cholesterol elimination and synthesis.

To test the effect of CYP27A1 deficiency on the development of atherosclerosis, we previously crossed C*yp27a1* knockout (KO) mice with Apolipoprotein E (*ApoE*) KO mice, and fed the offspring with a Western diet (WD) for 3 and 6 months [9]. The atherosclerotic phenotype in *ApoE* KO mice was reversed in *Cyp27a1/ApoE* double KO (DKO) whereas *Cyp27a1* heterozygote/*ApoE* KO (het) with reduced 27-OHC production developed much more severe lesions than *ApoE* KO mice. Low plasma cholesterol concentration and enhanced activities of hepatic sterol 7α-hydroxylase (CYP7A1) and sterol 12α-hydroxylase (CYP8B1), two cytochromes P450 involved in BAs formation, were main contributors to the protective effects in DKO mice. Additionally, increased expression of hepatic cytochrome P450 *Cyp3a11* involved in BA detoxification, also contributed to the antiatherosclerotic mechanism.

In humans, CYP3A4, the murine homolog of *Cyp3a11*, is known to metabolize more than 60 per cent of all therapeutic drugs. It accounts for the oxidative metabolism of compounds used for the treatment of hyperlipidemia (statins), bacterial infections (erythromycin), autoimmune diseases (cyclosporine), diabetes (thiazolidinediones and sulfonylureas), hypertension (calcium channel blockers), and cancer (cytostatics) [10,11]. CYP3A4 also metabolizes numerous endogenous chemicals. Thus, pharmacodynamic approaches modulating CYP3A4 activity offers an attractive option to target therapeutic effects of endo- or xenobiotics metabolized by CYP3A4 in humans. Rifampicin (RIF), often administered as an antibiotic for the treatment of tuberculosis, is a potent CYP3A4 inducer [12]. It exerts its activity via Pregnane X receptor (PXR) activation [13].

Based on our observations in DKO mice [9] and to investigate the relevance of CYP3A11 for cholesterol homeostasis, we analyzed the effect of *Cyp3a11* induction by RIF on atherosclerosis in *ApoE* KO and het mice with reduced *Cyp27a1* expression.

## Experimental

### Chemicals

RIF (R3501) was purchased from Sigma-Aldrich (St Louis, MO, U.S.A.). [4-^14^C] Cholesterol and [22,23-^3^H] β-sitosterol were obtained from American Radiolabeled Chemical, Inc. (St Louis, MO, U.S.A.). All other relevant chemicals are listed under ref. [9].

### Animals

Animal experimentation was approved by the Ethics Committee for Animal Experiments of the Veterinary Administration of the Canton of Bern, Switzerland, and conformed to the rules of the Swiss Federal Act on Animal Protection Guide for the Care and Use of Laboratory Animals.

*ApoE* KO mice and their het counterparts (C57BL/6) were bred from het X het mating. Pups were maintained and genotyped as previously published [9]. For the experiments, male *ApoE* KO and het were weaned at the age of 4 weeks and fed from this age with a WD containing 21% fat and 0.15% cholesterol (Provimi Kliba, Switzerland). At the age of 6 weeks, a first cohort of mice was divided in two groups (*n*=6) and treated daily with RIF (10 mg/kg ip) or vehicle (0.9% saline) for 4 weeks. At the end of the experiment, mice were starved for 4 h from 8 a.m. to 12 a.m. and killed by pentobarbital injection (300 mg/kg, pentobarbital sodium, USP; Abbott Laboratories, North Chicago, IL, U.S.A.) within the next 3 h. The animals were weighed and blood was collected into a tube containing 20–50 U heparin, centrifuged at 4°C for 15 min at 13000 rpm, and stored at −20°C until use. Organs were removed, one part was washed with PBS and fixed in formalin at 4°C, and the other part was frozen in liquid nitrogen and stored at −70°C. The heart and aorta were fixed in 4% paraformaldehyde for 24 h, transferred to PBS, and stored at 4°C.

A second cohort of *ApoE* KO and het mice (*n*=6) was fed at the age of 8 weeks with WD and cholesterol absorption was measured before and 2 weeks following daily injection of RIF (10 mg/kg ip) as described below.

### Characterization of atherosclerotic lesions

Lesions of the aortic valve were analyzed in paraffin embedded sections of 10 µm at 20 µm intervals for 700 µm and stained with hematoxylin and eosin (H&E) as described previously [9]. Lesion areas were quantified in a blinded manner by image analysis software (ImageJ; NIH, Bethesda, MD, U.S.A.).

### Biochemical analysis of plasma and liver

Triglycerides (TG), total cholesterol (TC), low-density lipoprotein-cholesterol (LDL-C), and high-density lipoprotein-cholesterol (HDL-C) were quantified in plasma with a kit from Wako Chemicals GmbH (Neuss, Germany). Glucose and alanine aminotransferase (ALT) were measured using an electrochemiluminescence immunoassay from Roche Diagnostics (Zug, Switzerland) respectively Elabscience (Chemie Brunschwig AG, Switzerland). In liver homogenates, TG was measured using a TG quantification kit (BioVision, Mountain View, CA, U.S.A.), cholesterol, and cholesteryl esters (CE) with a CE quantification kit (Calbiochem-Merck Millipore, Zug, Switzerland). 27-OHC was quantified in 100 µl of plasma by gas-chromatography and mass spectrometry (GC-MS). The same extraction procedure as the one described for HPLC-MS was used [14]. Samples were then derivatized with 100 µl of pyridine and 100 µl of N,O-bis-trimethylsilyl-trifluoroacetamide for 1 h at 60°C, using 5α-cholestan-3β,6α-diol and stigmasterol (100 ng each) as internal standard, and applied on GC-MS [9,15].

### RNA extraction and real-time PCR in liver and jejunum

RNA extraction and real-time PCR in liver and jejunum homogenates were conducted according to standard procedures as described [9]. Reverse transcription was performed with 2 µg of RNA in a reaction containing 100 U SuperScript Reverse Transcriptase type II (Roche, Basel, Switzerland). Real-time PCR was performed with 100 ng cDNA/reaction. TaqMan assay for *Abcg1* (Mm01348250_m1) was ordered from Life Technologies-Applied Biosystems (Switzerland). For Niemann-Pick C1-like 1(*Npc1l1*), forward and reverse primers (caacatcttcatctttgttcttgag and gccaatgtgagcctctcg) were from Microsynth (Switzerland) and UPL probes (number 110) from Roche Diagnostics (Germany). The list of primers and probes were described in the previous study [9]. *Actb* (AM1720) was used as the internal standard. Quantification was performed by the relative quantification method with samples from *ApoE* KO mice fed WD and treated with vehicle used for calibration.

### Cholesterol absorption

In a second cohort of mice, gavage was performed with 100 μl of soybean oil containing 0.1 μCi of [4-^14^C] cholesterol and 0.2 μCi of [22, 23-^3^H] β-sitosterol. After gavage, each mouse was individually housed in a cage covered with Whatman paper and had free access to food and water. Feces were collected daily for 3 days. Samples were extracted by saponification with 5 M KOH in 50% ethanol and heated at 80°C for 1 h. Lipids were extracted by Folch method. The extracted lipids were transferred into scintillation vials and isotopes were counted in β-counter. The percentage of cholesterol absorption was calculated as follows:

$$\frac{(14C/3H)dose\;ratio\;-\;(14C/3H)faeces\;ratio}{(14C/3H)dose\;ratio}\;\times \;100$$

### Statistical analysis

To determine statistically significant differences, ANOVA was used followed by *post hoc* test (Tukey or Bonferroni) for multiple comparisons.

## Results

### Effects of RIF on body and organs weight and liver histology

**Figure 1:**
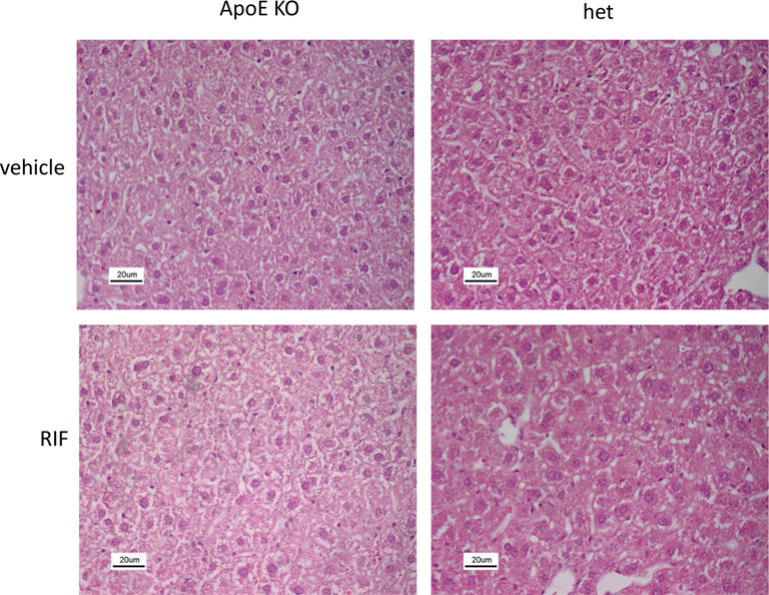
Effect of RIF on liver morphology Representative photomicrographs of H&E liver sections of *ApoE KO* and het mice fed with WD and treated with 10 mg/kg RIF or vehicle i.p. daily for 4 weeks; scale bars: 20 μm.

Administration of RIF to *ApoE* KO and het mice did not affect body weight, nor liver, spleen, kidney, and brain weights at all time points investigated (Table 1). Treatment with RIF had no effect on general morphology of the liver; no hepatotoxicity was observed under the conditions used (Figure 1), and hepatic transaminase (ALT) remained unchanged (Table 1).

**Table 1:** Effect of RIF on organs weights, plasma and hepatic parameters

	apoE KO		het		
	vehicle	RIF	vehicle	RIF	
Parameter	n = 6	n = 6	n = 6	n = 6	P value
Weight					
Body (g)	25.6±0.7	25.4±0.6	24.9±0.4	25.2±0.8	**NS**
Liver (g)	1.46±0.05	1.46±0.04	1.38±0.05	1.32±0.05	**NS**
kidney (g)	0.15±0.01	0.15±0.01	0.14±0.01	0.15±0.01	**NS**
Spleen (g)	0.11±0.01	0.12±0.02	0.13±0.02	0.12±0.03	**NS**
Brain (g)	0.46±0.01	0.45±0.01	0.44±0.01	0.43±0.01	**NS**
Plasma					
TC (mmol/l)	22.2±5.4	9.4±1.9 ^##^	18.0±2.1	17.6±1.0 ***	**< 0.0001**
LDL-C (mmol/l)	17.2±1.7	12.2±1.7 ^###^	11.7±1.44 ***	13.3±2.4	**0.0003**
HDL-C (mmol/l)	0.57±0.15	0.88±0.24 ^#^	0.57±0.14	0.53±0.14 *	**0.0217**
HDL/LDL-C	0.03±0.01	0.07±0.03 ^##^	0.05±0.01	0.04±0.01 *	**0.0018**
TG (mmol/l)	1.5±0.6	2.1±0.6	0.9±0.3	1.0±0.3**	**0.0002**
Glucose (mmol/l)	7.5±0.6	8.5±0.5	6.5±0.5	7.5±0.8	**NS**
ALT (ng/ml)	7.0±2.0	7.5±1.5	7.4±1.6	7.8±1.5	**NS**
Markers of CYP27A1 activity					
27-OHC (ng/ml)	459±177	323±114	179±46 **	274±54	**0.0073**
CYP27A1 activity (ng/mg)	54±17	91±41 ^#^	30±12*	40±8 *	**0.0351**
Liver					
TC (μg/mg)	11.3±1.7	8.3±1.0 ^##^	8.9±1.4 *	9.1±0.6	**0.0037**
CE (μg/mg)	5.1±1.4	5.0±1.0	3.6±0.8	4.5±0.7	**NS**
TG (nmol/mg)	32.8±8.7	40.0±7.5	36.1±11.9	33.0±16.4	**NS**

Results are presented as means ± SD. Significant differences were revealed by two-way ANOVA followed by Bonferroni's post-test, with # *P*<0.05, ## *P*<0.01, ### *P*<0.001 for the effect of RIF and * *P*<0.05, ** *P*<0.01, *** *P*<0.001 for the effect of the genotype

### Effect of RIF on development of atherosclerotic plaque

**Figure 2:**
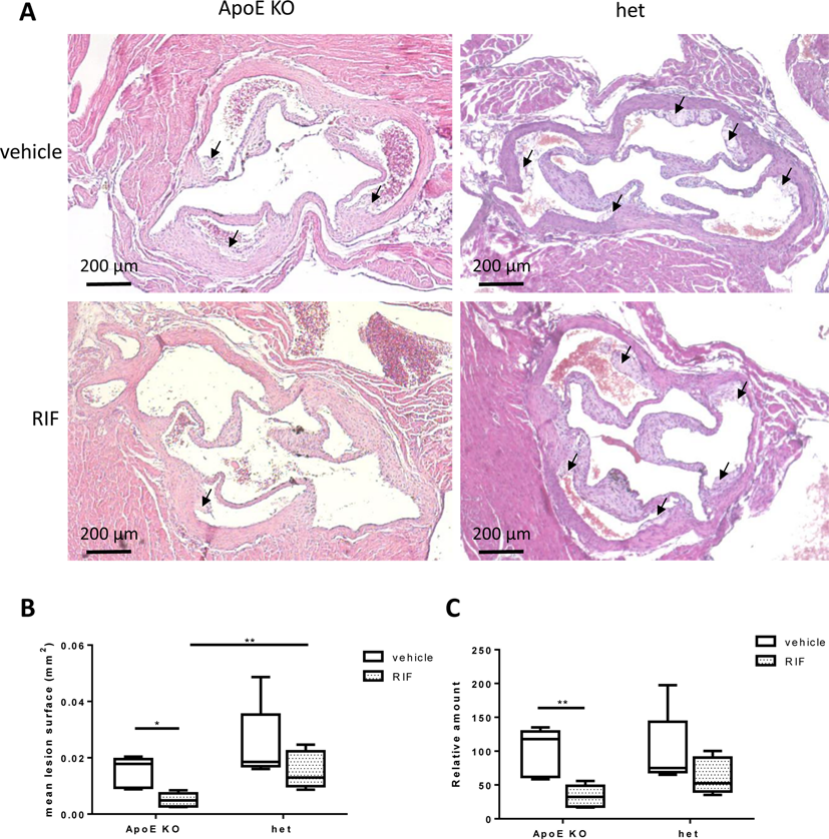
Effect of RIF on development of atherosclerosis Representative photomicrographs of aortic valve in paraffin-embedded sections stained with H&E in *ApoE KO* and het mice fed with WD and treated with 10 mg/kg RIF or vehicle i.p. daily for 4 weeks; scale bars: 200 μm. (**A**) Quantification of atherosclerotic lesions in [mm^2^] (**B**) and as relative amount of atherosclerotic plaque when compared with vehicle (**C**). Arrows indicate atherosclerotic plaques. Means with whiskers (minimum and maximum) are presented; *P<0.05, **P<0.01.

A representative picture of atherosclerotic lesions is shown in Figure 2A. Atherosclerotic lesions were significantly reduced upon RIF treatment in *ApoE* KO mice (0.013 ± 0.003 vs 0.005 ± 0.001 mm^2^, mean ± SEM, *P*<0.05) but not in het (0.022 ± 0.006 vs 0.015 ± 0.003, mean ± SEM, NS) (Figure 2B). The amount of atherosclerotic plaque tend to be higher in het than in *ApoE* KO mice, but the difference was significant only in the RIF group (0.015 vs 0.005 mm^2^, *P*<0.01) (Figure 2A and B). When the relative amount of atherosclerotic plaque was considered, RIF reduced atherosclerosis formation by 66% in *ApoE* KO mice and by only 38% in het (Figure 2C).

### Effect of RIF on plasma and liver

Next we analyzed plasma lipid and lipoprotein profile (Table 1). In *ApoE* KO mice, RIF treatment led to a 2-fold decrease in TC, a 1.5-fold decrease in LDL-C, and 1.5-fold increase in HDL-C; as a result HDL/LDL-C ratio increased 2.5-fold. There was no significant difference in TG levels. CYP27A1 activity, assessed as 27-OHC/TC ratio, was increased by RIF. Plasma glucose was unchanged (Table 1). In the liver, RIF significantly reduced TC but did not lead to any changes in cholesterol ester (CE) or TG in *ApoE* KO mice.

In het mice, RIF did not lead to significant changes in plasma lipids (Table 1). When compared with *ApoE* KO mice, after RIF treatment, het had higher TC levels and lower HDL-C, HDL/LDL-C ratio, TG, and CYP27A1 activity (Table 1). In het mice, RIF had no impact on liver lipids.

### Effect of RIF on gene expression in the liver

**Figure 3:**
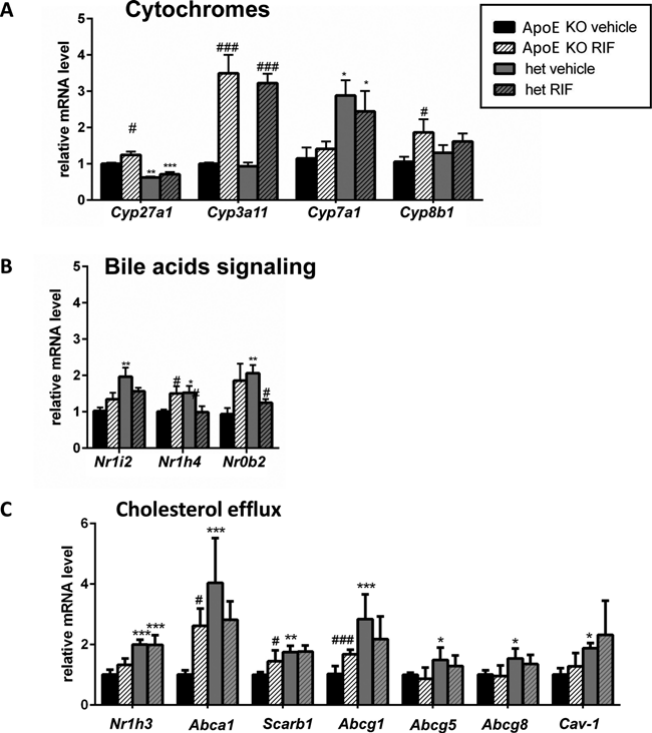
Effect of RIF on gene expression in the liver The expression of genes encoding for cytochromes P450 (**A**), BAs signaling (**B**), and cholesterol efflux (**C**) was quantified in *ApoE* KO and het mice fed with WD and treated with 10 mg/kg RIF or vehicle i.p. daily for 4 weeks, using β-actin as housekeeping gene. Results are presented as mean ± SEM; *P<0.05, **P<0.01, ***P<0.001, vs *ApoE* KO, ^#^*P*<0.05, ^###^*P*<0.001 vs vehicle.

The effect of RIF on the expression of genes of the cytochrome family, those involved in BAs signaling and cholesterol efflux, was analyzed in the liver (Figure 3). RIF led to a 4-fold increase of the expression of *Cyp3a11* in both genotypes. RIF also induced expression of *Cyp27a1* and *Cyp8b1* in *ApoE* KO but not in het mice and had no effect on *Cyp7a1* expression (Figure 3A). A genotype mediated effect of RIF was seen for the receptors involved in BA signaling (Figure 3B). RIF reduced the expression levels of *Nr1h4* (gene for farnesoid receptor (FXR)) and *Nr0b2* (gene for small heterodimer partner (SHP)) in het mice, but increased *Nr1h4* expression in *ApoE* KO mice. The analyses of the expression of genes involved in cholesterol efflux revealed that RIF induced the expression of *Abca1, Scarb1* (gene for scavenger receptor class B member 1), and *Abcg1* in *ApoE* KO mice (Figure 3C). In het mice, the expression of all the genes involved in cholesterol efflux was higher than in their *ApoE* KO littermates but did not change upon treatment with RIF (Figure 3C).

### Effect of RIF on intestinal cholesterol absorption and gene expression in jejunum

**Figure 4:**
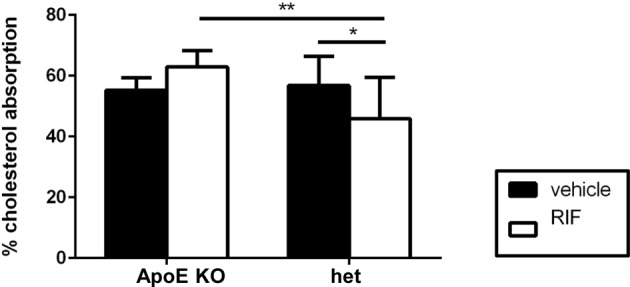
Effect of RIF on cholesterol absorption Cholesterol absorption was measured in *ApoE* KO and het mice fed with WD and following treatment with 10 mg/kg RIF or vehicle i.p. daily, using dual absorption method. Results are presented as mean ± SEM; *P<0.05, **P<0.01.

**Figure 5:**
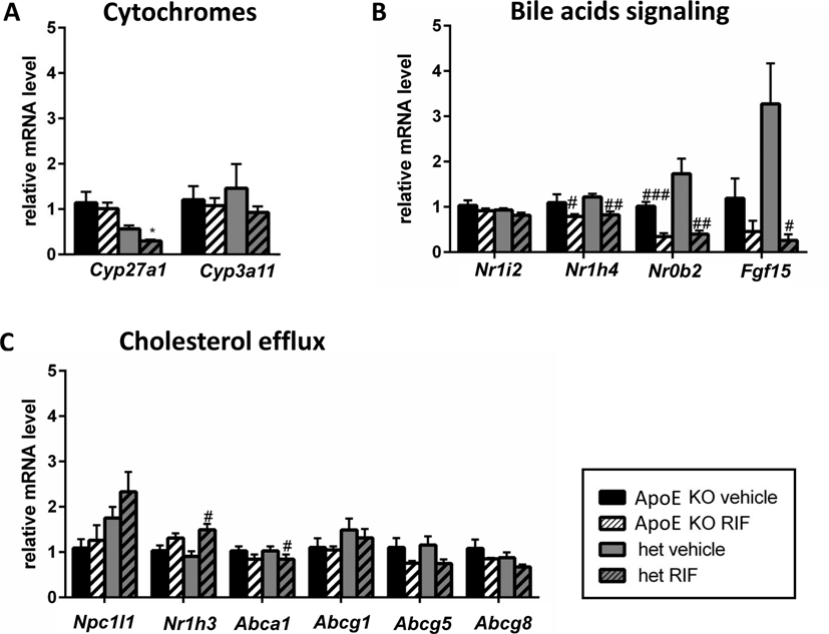
Effect of RIF on cholesterol homeostasis in the jejunum The expression of genes encoding for cytochromes P450 (**A**), BAs signaling (**B**), and cholesterol efflux (**C**) was quantified in *ApoE* KO and het mice fed with WD and treated with 10 mg/kg RIF or vehicle i.p. daily for 4 weeks using β-actin as housekeeping gene. Results are presented as mean ± SEM; *P<0.05, vs *ApoE* KO, ^#^*P*<0.05, ^##^*P*<0.01, ^###^*P*<0.001 vs vehicle.

Cholesterol absorption was assessed using the fecal dual-isotope ratio method (Figure 4). The same percentage of cholesterol absorption was observed in vehicle treated *ApoE* KO and het mice. RIF had no effect on cholesterol absorption in *ApoE* KO but significantly reduced it in het mice (*P*<0.05). To explore the mechanism accounting for the changes in cholesterol absorption, expression of cytochromes P450 (Figure 5A), genes involved in BAs signaling (Figure 5B), and cholesterol transport (Figure 5C) were analyzed in the intestine. RIF had no effect on *Cyp3a11* expression in the jejunum in *ApoE* KO or het mice but down-regulated *Cyp27a1* mRNA levels in het mice (Figure 5A). RIF reduced expression of *Nr1h4, Nr0b2*, and *fgf15* (fibroblast growth factor 15) to the same magnitude in both genotypes and had no effect on expression of *Nr1i2* (gene for PXR) (Figure 5B). In *ApoE* KO mice, RIF had no significant effect on the expression level of genes involved in cholesterol transport (Figure 5C). In het, RIF slightly induced *Nr1h3* (gene for LXR α) expression and slightly reduced *Abca1* expression.

## Discussion

In our previous study [9], DKO mice fed with WD developed 10-fold less atherosclerosis than their *ApoE* KO littermates. In contrary, het mice with reduced *Cyp27a1* expression had much more atherosclerosis than *ApoE* KO mice. Among the proposed protective mechanisms in DKO mice, increased cholesterol metabolism via overexpression of hepatic *Cyp3a11* was proposed. CYP27A1 was also atheroprotective, since het mice with reduced plasma 27-OHC concentration and normal levels of *Cyp3a11* expression developed more atherosclerotic lesions than *ApoE* KO mice. To investigate the influence of cholesterol metabolism by CYP3A11 and CYP27A1 on development of atherosclerosis, RIF, a known inducer of *Cyp3a11*, was administrated to *ApoE* KO and het mice.

**Figure 6:**
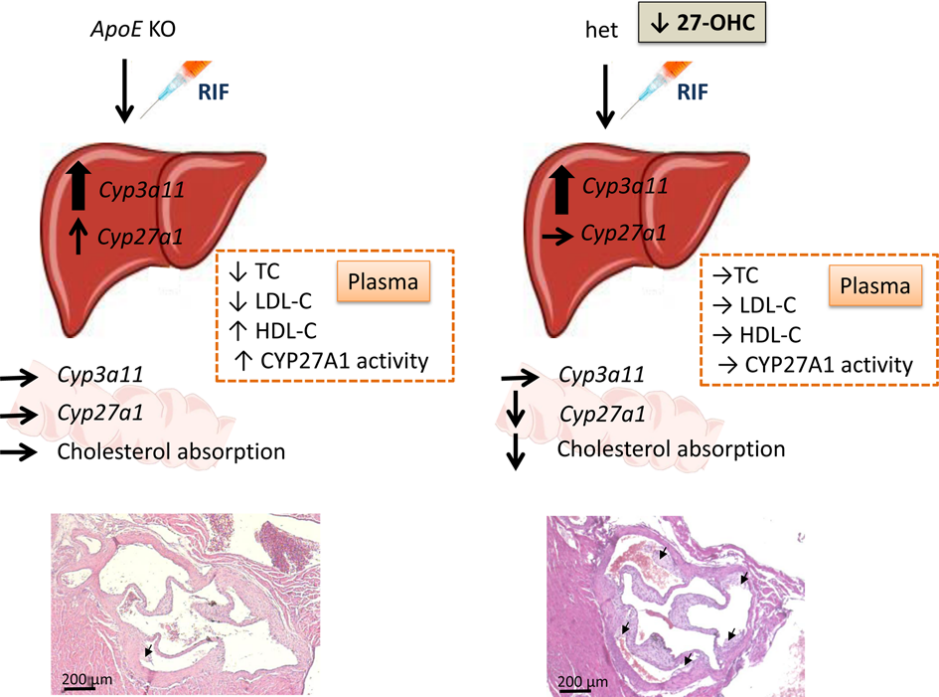
Cyp27a1 expression and the antiatherosclerotic effect of RIF in mice The effect of *Cyp3a11* induction by RIF on atherosclerosis development was analyzed in *ApoE* KO mice with normal *Cyp27a1* expression and 27-OHC plasma concentration, and in het mice with reduced *Cyp27a1* expression and 27-OHC plasma concentration. RIF induced hepatic *Cyp3a11* mRNA 3-fold and had no effect on intestinal *Cyp3a11* expression in both genotypes. In *ApoE* KO mice, RIF increased hepatic *Cyp27a1* mRNA, leading to an increase in CYP27A1 activity measured by the 27-OHC/Cholesterol ratio in plasma. RIF also decreased TC and LDL-C and increased HDL-C. In het mice, RIF reduced *Cyp27a1* mRNA levels in the intestine and had no effect on plasma lipids, despite reduced cholesterol absorption. The different effect of RIF on atherosclerosis development in *ApoE* KO and het mice underline the importance of *Cyp27a1* expression in the protection of atherosclerosis.

The key findings of our study can be summarized as follows (Figure 6). First, RIF protected *ApoE* KO mice fed a proatherogenic diet from atherosclerosis development. Second, RIF induced *Cyp3a11* hepatic expression to the same levels in *ApoE* KO and het mice. Third, RIF had a specific effect on CYP27A1 in *ApoE* KO mice, as demonstrated by (i) increased hepatic expression of *Cyp27a1* and (ii) elevated apparent CYP27A1 activity, measured as 27-OHC/TC ratio in plasma. Finally, RIF did not induce intestinal *Cyp3a11*. In *ApoE* KO mice, RIF had no influence on cholesterol absorption. In contrary in het mice, RIF slightly reduced the expression of *Cyp27a1* and cholesterol absorption. These observations point to the importance of an adequate expression of *Cyp27a1* to protect against atherosclerosis development *in vivo* in mice.

The antiatherosclerotic effect of RIF in *ApoE* KO mice was likely due to its effect on plasma lipids and lipoproteins. Favorable changes in lipid profile consisting in a decrease in plasma TC and LDL-C combined with an increased in plasma level of HDL-C and increased CYP27A1 activity, is a likely reason for which *ApoE* KO mice treated with RIF developed less atherosclerosis than their vehicle-treated littermates. RIF also significantly increased the expression of *Cyp27a1, Abca1, Scarb1*, and *Abcg1* in the liver, all four genes involved in cholesterol efflux. The effect of RIF on development of atherosclerosis was less pronounced in het mice with reduced *Cyp27a1* expression. The plasma proatherogenic properties, such as high TC, high LDL-C, low HDL-C, and low apparent CYP27A1 activity, remained unchanged upon treatment with RIF in het mice. Furthermore, the expression of hepatic genes involved in cholesterol efflux was not induced by RIF in het mice. *Cyp27a1* expression was slightly decreased in the intestine, but the decrease in intestinal cholesterol absorption was not sufficient to decrease atherosclerotic plaque to the level of this observed in *ApoE* KO mice. Based on these findings, we conclude that, first, the differences in the effect of RIF on development of atherosclerosis was mainly due to the level of *Cyp27a1* expression in the liver and, second, that RIF treatment was less effective when *Cyp27a1* expression was reduced.

Our findings on lipoprotein profile changes in plasma of *ApoE* KO mice are consistent with a previous study from Bachmann et al. [16]. In this work, induction of Cyp3a11 with imidazole in rats and with RIF in mice led to increased levels of plasma HDL-C and apoA-1. The effect was mediated by PXR, since similar treatment in wild-type and *Pxr* KO mice had a different effect on plasma HDL-C and apoA-I [16].

Although the effect of RIF on human PXR is well established, we have shown in the present study that RIF can also increase *Cyp3a11* expression in non-humanized mice. This result contrasts with several reports that failed to show any induction of *Cyp3a11* in the liver. This discrepancy can be explained as follows. First, much shorter treatments (4–5 days) were used in these studies [17–19]. Second, the mode of administration differed. Since we applied RIF i.p. and not by gavage, the effect of gastric acid on the drug was avoided. Finally, the genetic background of the animals was different; Hosagrahara et al. [18] used BALB/c mice, while our studies were performed in C57BL/6 mice.

Enhanced expression of *Cyp27a1* in addition to the induction of *Cyp3a11* expression is an interesting finding, given the role of CYP27A1 in cholesterol efflux [5]. In het mice with reduced CYP27A1 activity, the effect of RIF on hepatic *Cyp27a1* was less pronounced, and plasma level of TC was higher. Our results are in line with those of Sheng et al. [20] who described a direct effect RIF on hepatic *Cyp27a1* mRNA expression in ICR mice. A 3 weeks treatment with 100 mg/kg/day led to an increase in serum 25-hydroxy vitD_3_, a metabolite obtained from the conversion of vit D_3_ by CYP27A1 in the liver.

Whether our findings can be extrapolated to humans remains to be seen, given the profound dissimilarities between bile acids metabolism between humans and mice. Indeed, the phenotype of Cyp27a1 KO mice was mainly expressed in the liver, with a severe microvesicular steatosis, whereas CYP27A1 deficiency in humans with Cerebrotendinous Xanthomatosis (CTX), clinical manifestations include bilateral juvenile cataracts and accumulation of cholestanol in different tissues [21]. For many years, RIF was used as an antibiotic for the treatment of tuberculosis and it is still used in clinical practice [12]. CTX, patients, induction of CYP3A4 activity by RIF cannot replace the effective therapy with chenodeoxycholic acid (CDCA). Whereas CDCA treatment leads to the inhibition of cholesterol and 7α-hydroxycholesterol synthesis and a reduction in plasma cholestanol concentration, upon RIF treatment, cholestanol levels remain unchanged, despite a 60% increase in CYP3A4 activity [22]. The difference of effect between the two substances could be explained, at least in part, by the missing effect of RIF on CYP27A1, in the absence of CYP27A1 expression in CTX patients. In healthy volunteers, daily treatment with 600 mg RIF has no effect on normal plasma cholesterol at first, but after a week, it increases cholesterol steadily [23]. The same dose of RIF induces hypercholesterolemia in patients with acute brucellosis treated with a bitherapy consisting of RIF (600 mg/day) and Doxycycline (100 mg/day) [24]. Thus, in humans, treatment with RIF may not be as beneficial as in mouse models. However, in these studies, 27-OHC (which we use as marker for CYP27A1 activity to calculate the 27-OHC/cholesterol ratio) was not measured, making mechanistic conclusions about the effect of RIF on lipoprotein profile in humans difficult.

There are some limitations in the present study. The effect of RIF on atherosclerosis development was performed on young mice, at an early stage of atherosclerosis development. Whereas this remains true in elderly mice with more severe atherosclerotic lesions, remains to be proven. The length of the treatment was also relatively short. In order to avoid hepatotoxicity, RIF was administered for 4 weeks only. This is a relative short time frame for atherosclerosis development, even in the *ApoE* KO model with WD. Although we observed a marked induction of *Cyp3a11* in both genotypes, the level of increase was not related to PXR activation, since our mice were not crossed with PXR humanized mice. Finally, the mechanisms behind the observed effects remains uncertain. A putative mechanism, based on available data, is depicted in Figure 6. Further studies, such as knocking down *Cyp3a11* in *ApoE* KO and het mice would largely contribute to the clarification of the role of *Cyp27a1* and *Cyp3a11* in atherosclerosis development.

In conclusion, despite a similar induction level of *Cyp3a11* expression in the liver, RIF had a different effect on plasma lipids and atherosclerosis development in young *ApoE* KO and het mice, revealing the importance of *Cyp27a1* gene dosage *in vivo* in mice.

## Abbreviations

27-OHC: 27-hydroxycholesterol
ABCA1: ATP-binding cassette A1
ABCG1: ATP-binding cassette G1
ACTB: Actin
ApoA-1: apolipoprotein A-I
ApoE: apolipoprotein E
BA: bile acid
CDCA: chenodeoxycholic acid
CE: cholesteryl ester
CTX: cerebrotendinous xanthomatosis
CYP3A11: cytochrome P450 3A11
CYP27A1: sterol 27-hydroxylase
CYP7A1: cytochrome P450 enzyme 7α-hydroxylase
CYP8B1: sterol 12-alpha-hydroxylase
DKO: *Cyp27a1* KO/*ApoE* KO mice
FGF15: fibroblasts growth factor 15
FXR: farnesoid X receptor
GC-MS: gas chromatography - mass spectrometry
HDL-C: high-density lipoprotein cholesterol
H&E: hematoxylin and eosin
Het: *Cyp27a1* heterozygote/*ApoE* KO mice
HMGCR: 3-hydroxy-3-methylglutaryl-CoA reductase
KO: knockout
LDL-C: low-density lipoprotein cholesterol
LDLR: low-density lipoprotein receptor
LXR: liver X receptor
NPC1L1: Niemann-Pick C1-like protein 1
PXR: pregnane X receptor
RCT: reverse cholesterol transport
RIF: Rifampicin
SHP: small heterodimer partner
TG: triglyceride
WD: Western diet
